# Rapid Screening and Identification of BSA Bound Ligands from *Radix astragali* Using BSA Immobilized Magnetic Nanoparticles Coupled with HPLC-MS

**DOI:** 10.3390/molecules21111471

**Published:** 2016-11-05

**Authors:** Liangliang Liu, Juan Leng, Xiai Yang, Liping Liao, Yin Cen, Aiping Xiao, Lei Ma

**Affiliations:** 1 Institute of Bast Fiber Crops, Chinese Academy of Agricultural Sciences, Changsha 410205, China; liuliangliang@caas.cn (L.L.); juanlengcaas@yahoo.com (J.L.); xiaiyang@yahoo.com (X.Y.); lipingliaocaas@yahoo.com (L.L.); 2 State Key Laboratory of Cotton Biology, Institute of Cotton Research of CAAS, Anyang 455000, China; 3 College of Chemistry and Chemical Engineering, Central South University, Changsha 410083, China; hn_cenyin@csu.edu.cn

**Keywords:** bovine serum albumin, HPLC-MS, ligands, magnetic nanoparticles, *Radix astragali*

## Abstract

*Radix astragali* is widely used either as a single herb or as a collection of herbs in a complex prescription in China. In this study, bovine serum albumin functionalized magnetic nanoparticles (BSA-MN) coupled with high performance liquid chromatography-mass spectrometry (HPLC-MS) were used to screen and identify bound ligands from the *n*-butanol part of a *Radix astragali* extract. The prepared BSA-MN showed sufficient magnetic response for the separation with an ordinary magnet and satisfied reusability. Fundamental parameters affecting the preparation of BSA-MN and the screening efficiency were studied and optimized. Under the optimum conditions, four bound ligands were screened out from the *n*-butanol part of a *Radix astragali* extract and identified as genistin (**1**), calycosin-7-*O*-β-d-glucoside (**2**), ononin (**3**) and formononetin (**4**). This effective method could be widely applied for rapid screening and identification of active compounds from complex mixtures without the need for preparative isolation.

## 1. Introduction

Chinese herbal medicine has been widely used in China and many parts of Asia, and has a long history [1]. Chinese herbal medicine showed a relatively high medicinal value during the research because of the long history of usage among people, the various medical effects and low side effects [2]. Chinese herbal medicine also played an important role in drug discovery. More than sixty percent of drugs on the market were made from natural sources [3]. However, with the developments in modern medicine, there were still some problems in illustrating activity mechanisms of Chinese herbal medicine. Chinese herbal medicine was very complex and contained large amounts of hydrophobic and hydrophilic compounds [4]. The discovery of active compounds in Chinese herbal medicines is difficult due to its complexity [5]. The conventional bioassay guided fractionation of Chinese herbal medicines is a time consuming, labor intensive and low efficiency strategy [6]. The existence of these problems seriously hindered the research of new drugs [7]. Therefore, research on seeking biologically active compounds from Chinese herbal medicines was calling for a more efficient approach. In recent decades, many technologies like filtration, functionalized column, solid phase extraction, hyphenated instruments and virtual screening were focused on the screening and analysis of active compounds in various herbal medicines including Chinese red yeast rice, *Pueraria lobata*, *Saposhnikovia divaricata*, *Smilax glabra*, and *Oroxylum indicum* [8,9,10,11,12,13].

Recently, magnetic nanoparticles (MN) have aroused great interest in many biological and medicinal fields, principally because of its higher surface area, lower mass transfer resistance and ability to be more easily separated from the matrix by an external magnet [14,15,16]. The separation of MN using an ordinary magnet was rapid and convenient compared with the filtration and centrifugation. This kind of nanomaterial could be coated with inorganic or organic compounds to increase the stability for protecting against oxidation and aggregation [17,18]. The modification of MN also made it suitable for various kinds of applications. Because of its particular physical and chemical properties, MN was widely utilized in catalysis, solid phase extraction, active compound screening and biomolecule immobilization [19,20,21,22,23,24,25,26].

*Radix astragali* is one of the most widely used Chinese herbs present either as a single herb or as a collection of herbs in a complex prescription [27]. Pharmacological studies and clinical practice have demonstrated that *Radix astragali* possesses many biological functions [28,29]. It was used for the treatments of nephritis, diabetes, hypertension, cirrhosis and cancer in china [30]. Nowadays, *Radix astragali* is not only a traditional medicine but also a healthy food supplement. Various commodities containing *Radix astragali* are widely available [31]. Nevertheless, systematic research on the bindings between protein and constituents from *Radix astragali* is still in demand. Because of the abundance and important role in the blood circulatory system, serum albumin played an important role in drug delivery due to its remarkable binding property. The interaction between serum albumin and small molecules could result in a stable complex [32]. This kind of interaction attracted great interest and could be considered as a method for screening active compounds from complex samples. Due to the advantages of being low cost, easily available, having a good transport ability (including many endogenous and exogenous ligands) and high structure similarity to human serum albumin (HSA), bovine serum albumin (BSA) was one of the most extensively studied model proteins for exploring protein-ligand interactions [33,34].

In this study, BSA was selected as the target and functional molecule. BSA was functionalized on MN (BSA-MN) and the prepared material was utilized in the screening and identification of BSA bound ligands from *Radix astragali* coupled with high performance liquid chromatography-mass spectrometry (HPLC-MS). The results indicated that this method could rapidly screen active compounds from *Radix astragali* without the need of purification.

## 2. Results and Discussion

### 2.1. Characterizations of BSA-MN

**Figure 1:**
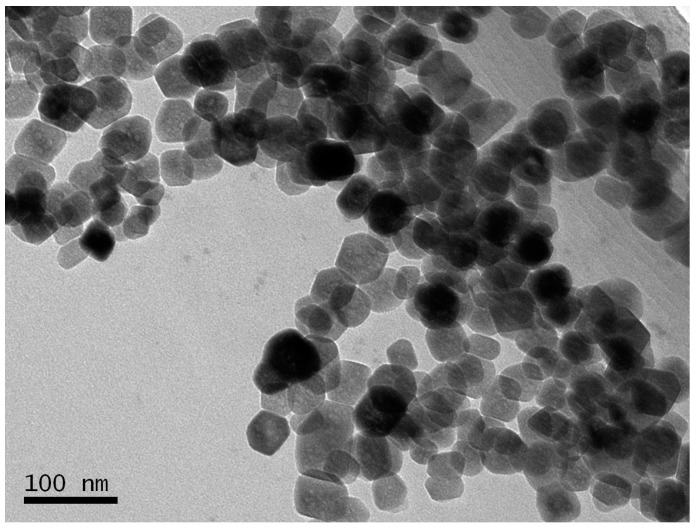
The TEM image of MN.

**Figure 2:**
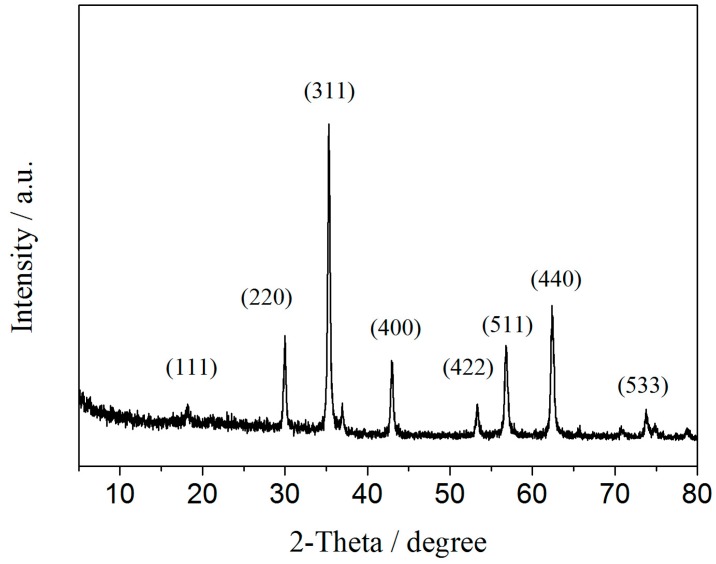
The XRD pattern of MN.

Figure 1 showed the TEM image of BSA-MN. The TEM image illustrated that the average diameter of MN was about 35 nm. The shape and size of MN were in accordance with the reported values [35]. The XRD pattern of MN was shown in Figure 2. It showed the peaks associated with their indices (111), (220), (311), (400), (422), (511), (440) and (533), respectively [18]. The peaks also agree with the standard Fe_3_O_4_ XRD spectrum (JCPDS Card 88-0866) [36].

**Figure 3:**
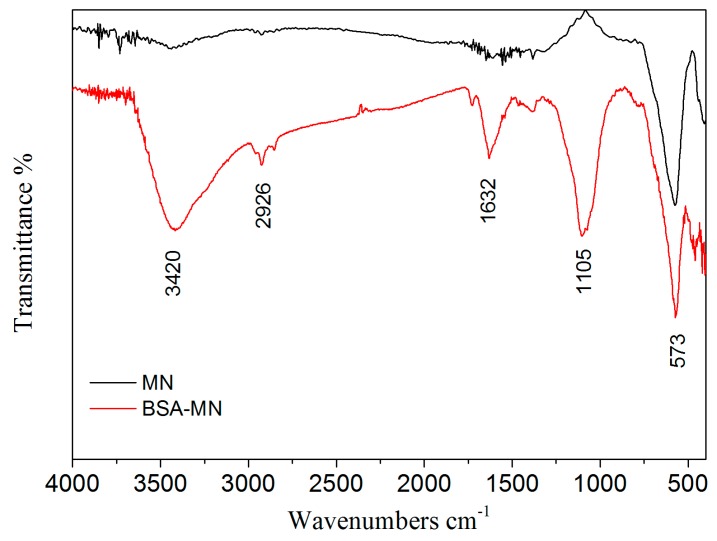
The FT-IR spectrum of MN (**black**) and BSA-MN (**red**).

Figure 3 showed the FT-IR spectrum of MN and BSA-MN. The adsorption peak at 573 cm^−1^ observed in the FT-IR spectrum of MN and BSA-MN was the characteristic absorption of the Fe-O bond, which confirmed the presence of MN. The spectrum of BSA-MN showed the stretching vibration peak observed at 3420 cm^−1^ could be attributed to the presence of N-H stretching of protein. The peak observed at 2920 cm^−1^ indicated the presence of C-H stretching of methyl group [37]. The peak at 1632 cm^−1^ was attributed to the presence of peptide bond formation between free single bond CHO group of glutaraldehyde and the amide group of protein [37]. The peak at 1105 cm^−1^ indicated the C-N bond presented in the amide bond [38]. These adsorption peaks indicated that BSA was immobilized on MN.

**Figure 4:**
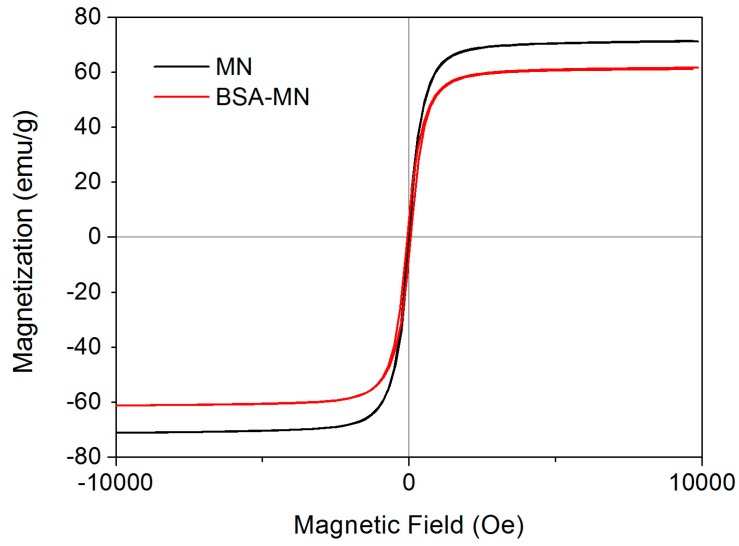
The magnetization curves of MN (**black**) and BSA-MN (**red**).

Magnetization curves of MN and BSA-MN were shown in Figure 4. The maximum saturation magnetizations of MN and BSA-MN were 71.4 and 61.0 emu/g, respectively. The maximum saturation magnetizations of BSA-MN were less than that of MN because of the existence of non-magnetic materials on the surface [39]. Due to the sufficient magnetic response, magnetic separation of BSA-MN with an ordinary magnet could be accomplished.

The immobilization amount of BSA was investigated by measuring the fluorescent intensity of supernatant solution after immobilization. As a result, the amount of BSA immobilized on MN was about 202 μg/mg after calculation.

### 2.2. Optimization of Immobilization Conditions

**Figure 5:**
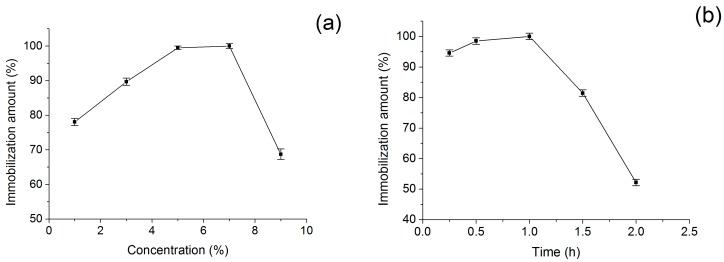
(**a**) Effect of glutaraldehyde concentration on immobilization when the activation time was 1.0 h; and (**b**) effect of activation time on immobilization when the glutaraldehyde concentration was 7%. The following immobilization was completed as the addition of BSA solution (2.0 mL, 1.0 mg/mL) and incubation for 4.0 h.

Glutaraldehyde is one of the most frequently used crosslinking agents in the biomolecular immobilization for the supports containing amino groups [40]. However, glutaraldehyde is a very versatile reagent, and its amount should be controlled. Considering the immobilization of BSA on MN was firstly activated with glutaraldehyde in this study, both glutaraldehyde concentration and activation time were investigated as important factors in the preparation of BSA-MN with a high amount of immobilized protein [41]. Figure 5 showed the effects of glutaraldehyde concentration and activation time on the immobilization. According to Figure 5a, the highest BSA immobilization amount could be observed when the glutaraldehyde concentration was 7%. It could be seen in Figure 5b that the BSA immobilization amount was increased as the activation time increased. The maximum BSA immobilization amount was found at an activation time of 1.0 h. With the extension of the activation time, the BSA immobilization amount was declined. Activation time shorter than 1.0 h led to a low BSA immobilization amount because of insufficient reaction between crosslinking reagent and amino acids, whereas longer activation time caused the loss of protein might be due to unnecessary crosslinking and diffusion limitations caused by increasing proteins on nanomaterial surfaces [41,42,43]. As a result, the optimum activation conditions were determined to be activated with 7% glutaraldehyde for 1.0 h.

**Figure 6:**
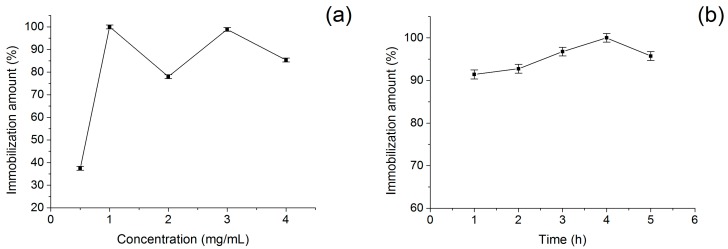
(**a**) Effect of BSA concentration on immobilization when the immobilization time was 4.0 h; and (**b**) effect of immobilization time on immobilization when the BSA concentration was 1.0 mg/mL. The previous activation was completed as the addition of glutaraldehyde solution (1.0 mL, 7% *w*/*v*) and incubation for 1.0 h.

BSA would be immobilized onto MN after an activation procedure. In order to obtain the optimum immobilized condition, experiments with different BSA concentration and immobilization time were investigated as well. In Figure 6a, the fluorescent intensity of both initial BSA solution and final supernatant solution after immobilization were detected and the difference between them could be considered as the BSA immobilization amount. Increment on the BSA immobilization amount was expected with increasing of initial BSA concentration. It was observed that BSA immobilization amount increased as the BSA concentration increased from 0 to 1.0 mg/mL. However, increase in the BSA concentration from the 1.0 to 4.0 mg/mL showed some declines in BSA immobilization amount. This might be due to the saturation of BSA on nanomaterial surfaces and the aggregation of MN. This kind of trend showed agreement with much of the reported literature [44,45]. Although the BSA immobilization amount at 3.0 mg/mL was close to that at 1.0 mg/mL, relatively low BSA concentration value would be chosen as the optimum value in consideration of the economy. Therefore, the optimum BSA concentration in immobilization was selected as 1.0 mg/mL. Figure 6b showed the effects of immobilization time on immobilization. When the immobilization time was 4.0 h, the BSA immobilization amount was the highest. Therefore, the optimum immobilization time could be determined as 4.0 h.

### 2.3. Optimization of Screening Conditions

**Figure 7:**
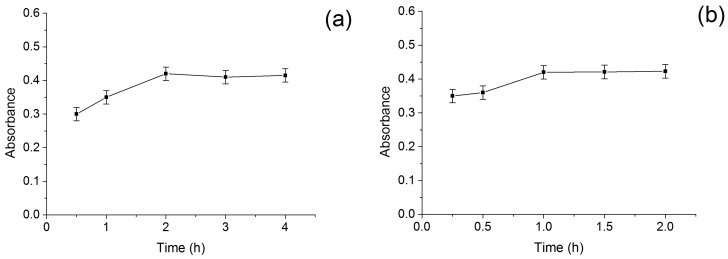
(**a**) Effect of incubation time on screening; and (**b**) effect of eluting time on screening.

The interaction between BSA and extract needs a certain concentration of BSA and enough incubation time in order to screen the bound ligands thoroughly. After screening, the BSA-MN was washed and dispersed in methanol solution to elute the ligands bound to BSA due to higher solubility. Figure 7 showed the effects of incubation time (Figure 7a) and eluting time (Figure 7b) on the screening. According to the experiment results, the absorbance of eluents reached its highest when the incubation time was 2 h and the eluting time was 1 h, respectively. In addition, the absorbance kept steady with the time prolonged. Therefore, the optimum incubation and eluting time were considered as 2 h and 1 h, respectively.

### 2.4. Screening and Identification of Bound Ligands from Radix astragali Extract

**Figure 8:**
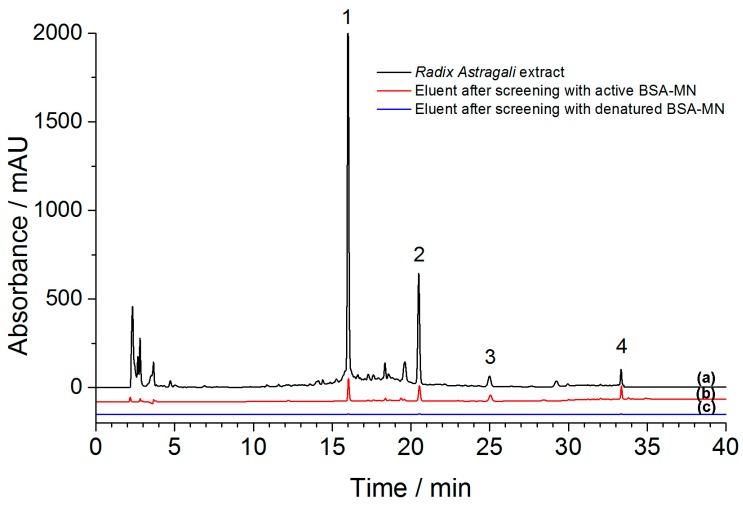
The chromatograms of (**a**) *n*-butanol part of *Radix astragali* extract (**black**); (**b**) eluent after screening with active BSA-MN (**red**) and (**c**) eluent after screening with denatured BSA-MN (**blue**).

Nonspecific adsorption between compounds and BSA should be excluded in order to ensure the accuracy of experiment results. Therefore, screening using denatured BSA-MN was accomplished in this study together with the screening using active BSA-MN. Figure 8a showed the chromatogram of *n*-butanol part of the *Radix astragali* extract, while Figure 8b,c showed the chromatograms of eluent after screening with active and denatured BSA-MN. Compared with the chromatogram of *n*-butanol part of the *Radix astragali* extract, no peak was observed in the chromatogram of eluent after screening with denatured BSA-MN. Apparently, four peaks marked with numbers appeared in the chromatogram of eluent after screening with active BSA-MN. Thus, four BSA bound ligands were screened out by BSA-MN combined with HPLC.

**Figure 9:**
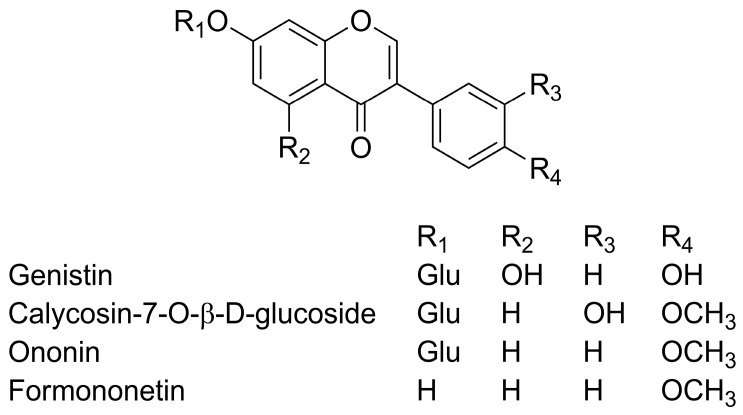
The chemical structures of four investigated compounds.

The chemical structures of four compounds were identified by HPLC-MS experiment. According to the data of their retention times, UV adsorptions and MS fragments shown in Table 1, the identifications of four compounds were confirmed and their structures were shown in Figure 9. Based on the analysis of UV spectra, all four of the compounds exhibited two absorbance bands at about 260 nm and 300 nm. This kind of adsorption were accorded with the typical spectra of isoflavone derivatives. Deprotonated molecular ion of compounds [M + H]^−^ could be observed in the MS spectra in positive mode. The [M − 162 + H]^−^ ion in MS spectra could be considered as the existence of glucoside. The [M − 15 + H]^−^ ion observed in the fragments was attributed to the neutral loss of methyl. Through analyzing the UV absorbance and MS spectrum of each compound, four compounds were identified as genistin (**1**), calycosin-7-*O*-β-d-glucoside (**2**), ononin (**3**) and formononetin (**4**). These data were in agreement with literature values and these compounds showed various activities in research [46,47,48,49,50].

According to the reported references, all four of these compounds showed binding activities with BSA, and the binding constant (log *K*a) and binding sites (*n*) of genistin, calycosin-7-*O*-β-d-glucoside and formononetin were calculated. As reported by Xiao, the log *K*a values of genistin and formononetin at 310 K were 5.1580 and 3.40. Moreover, the *n* values of them were 1.170 and 0.75, respectively [3,51]. As reported by Liu, the values of *K*a and *n* of calycosin-7-*O*-β-d-glucoside at 310 K were 0.15286 × 10^5^ and 0.9376 [52]. As reported by Wen, the interaction of a prescription of Danggui Buxue Decoction with BSA was studied by microdialysis coupled with HPLC-DAD-MS and nine compounds were identified. As a result, ononin and calycosin-7-*O*-β-d-glucoside showed binding activities. The binding degrees of them were 36.8% and 39.8%, respectively [53]. This information proved that the proposed screening method using BSA-MN for BSA bound ligands from natural products was efficient and effective.

### 2.5. Reusability of BSA-MN

**Figure 10:**
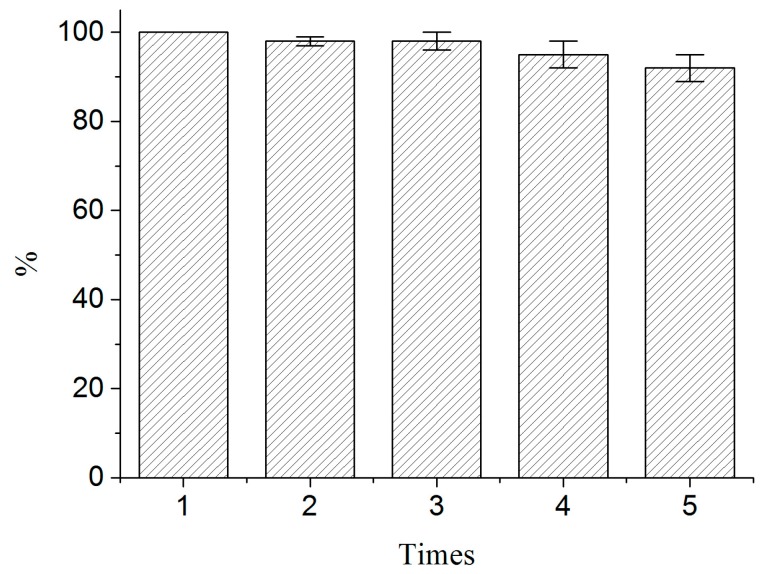
Reusability of BSA-MN.

BSA-MN was recycled and reused to allow continuous screening of active compounds [18]. After screening and elution, the supernatant was removed for analysis. The reusability of BSA-MN was investigated by screening sample with the recycled BSA-MN. As shown in Figure 10, the performance of BSA-MN decreased a little after screening five times, while no significant decrease of absorbance was found. These results indicated that the BSA-MN exhibited satisfying reusability in screening.

## 3. Materials and Methods

### 3.1. Materials

*Radix astragali* was purchased from Hunan Sanxiang Chinese Medicine Pieces Co., Ltd. (Changsha, China) BSA and glutaraldehyde (25% *w*/*v* aqueous solution) was purchased from Sinopharm Chemical Reagent Co., Ltd. (Shanghai, China). Acetonitrile was HPLC grade from Tedia Company Inc. (Phoenix, AZ, USA). Ultrapure water (18.2 MΩ·cm resistivity) was obtained from a Milli-Q water purification system (Millipore, Bedford, MA, USA). All of the other chemicals were analytical grade and purchased from Sinopharm Chemical Reagent Co., Ltd. (Shanghai, China). Genistin, calycosin-7-*O*-β-d-glucoside, ononin and formononetin were purchased from Shanghai Yuanye Biotechnology Co., Ltd. (Shanghai, China). The purity of each compound was determined to be ≥98% by HPLC analysis.

### 3.2. Synthesis and Characterization of BSA-MN

Firstly, ferric chloride (1.20 g) was dissolved in ethylene glycol (40.0 mL) to form a clear solution, followed by the addition of anhydrous sodium acetate (3.60 g) and 1,6-hexanediamine (3.60 g). The mixture was stirred vigorously for 30 min and then sealed in a Teflon lined stainless steel autoclave. The autoclave was heated at 180 °C for 6 h. The black products were then washed several times with ethanol and dried in a vacuum oven at 50 °C.

The immobilization of BSA was performed as the following: MN (25 mg) was suspended in glutaraldehyde solution (1.0 mL, 7% *w*/*v*) and shaken for 1 h. After reaction, the activated MN was obtained by magnetic separation and washed three times with phosphate buffer (10 mM, pH 7.4). Then, BSA solution (2.0 mL, 1.0 mg/mL) was added and the solution was shaken for 4 h, after which the supernatant was removed through magnetic separation and the BSA-MN was washed with phosphate buffer three times and stored at 4 °C.

In order to further investigate the optimum immobilization conditions of BSA-MN, different glutaraldehyde concentrations (1%, 3%, 5%, 7% and 9%, *v*/*v*), activation times (0.25, 0.5, 1.0, 1.5 and 2.0 h), BSA concentrations (0.5, 1.0, 2.0, 3.0 and 4.0 mg/mL) and various immobilization times (1.0, 2.0, 3.0, 4.0 and 5.0 h) were investigated. After reaction, the supernatant BSA solution was removed by a magnet and the fluorescent intensity of supernatant solution was measured using a fluorescence spectrophotometer (FL-4600, Hitachi, Tokyo, Japan). The excitation wavelength was set at 280 nm and the fluorescent intensity at 340 nm was measured. After calculations, the immobilization amount of BSA was expressed as a relative form (%) with the maximal value set as 100%.

BSA-MN was characterized with a JEM-2100F transmission electron microscope (JEOL, Tokyo, Japan) for TEM images. X-ray diffraction (XRD) was investigated on a Rigaku RINT 2500 powder X-ray Diffractometer (Rigaku Corporation, Tokyo, Japan). FT-IR spectra were obtained using a Nicolet avatar 360 FT-IR spectrophotometer (Thermo Fisher Nicolet, Orlando, FL, USA). Magnetization was recorded on a vibration sample magnetometer (VSM) VSM7307 (Lake Shore, Westerville, OH, USA) at room temperature.

### 3.3. Preparation of Radix astragali Extract

The *Radix astragali* (30.0 g) was extracted with ethanol solution (90% *v*/*v*, 300 mL) under reflux for 3 h. The solvent was concentrated under reduced pressure to yield a dried residue (1.38 g), which was further dissolved in 100 mL water and filtered through a 0.45 μm membrane (Acrodisc^®^ Syringe Filter, Pall, Ann Arbor, MI, USA). The aqueous solution was successively extracted with the same volume of petroleum ether, ethyl acetate and *n*-butanol according to the polar order. After that, three parts were evaporated to remove the solvents and obtain residues (petroleum ether part: 0.60 g, ethyl acetate part: 0.58 g and *n*-butanol part: 2.03 g), respectively. The *n*-butanol part of *Radix astragali* extract was dissolved in 100 mL of water and filtered by a 0.45 μm membrane. The solution was finally stored at 4 °C for further experiments.

### 3.4. BSA Bound Ligands Screening

BSA-MN (10 mg) was mixed with *n*-butanol part of *Radix astragali* extract (3.0 mL, 13.8 mg/mL). The mixture was shaken at room temperature for 2 h and separated by a magnet. BSA-MN was washed three times with phosphate buffer, and then methanol solution (3.0 mL) was added and shaken for 1 h to elute the ligands bound to BSA. Finally, the supernatant was stored at 4 °C for analysis.

In order to obtain the optimum screening conditions, experiments with different incubation times (0.5, 1, 2, 3 and 4 h) and eluting times (0.25, 0.5, 1, 1.5 and 2 h) were investigated. After experiments in these different conditions, the eluent was analyzed by a UV-2450 UV-VIS Spectrophotometer (Shimadzu, Kyoto, Japan) and the absorbance was measured at 254 nm. A higher absorbance denotes a higher affinity amount of bound ligands to BSA.

For the reusability test, the supernatant was removed for analysis after screening and elution. The residuals were washed with phosphate buffer three times and poured into another sample solution for next assay. The activity of BSA-MN in terms of the amount of bound ligands screened was evaluated through the UV detection at 254 nm.

### 3.5. HPLC-MS Analysis

The Dionex Ultimate 3000 HPLC (Dionex, Sunnyvale, CA, USA) and reversed phase SunFire C_18_ column (250 mm × 4.6 mm i.d., 5 μm, Waters, Milford, MA, USA) were employed for HPLC analysis. The mobile phase consisted of solvent A (water containing 0.4% *v*/*v* acetic acid) and solvent B (acetonitrile containing 0.4% *v*/*v* acetic acid) with gradient elution mode: 0–5 min, 10% B; 5–15 min, 10%–30% B; 15–25 min, 30% B; and 25–35 min, 30%–80% B. The flow rate was 1.0 mL/min and the column temperature was controlled at 30 °C. Spectra were recorded from 190 to 400 nm while the chromatogram was acquired at 254 nm.

The supernatant was analyzed by HPLC-MS for identification of bound ligands. HPLC analysis was performed on an Agilent 1290 Infinity LC System (Agilent Technologies Inc., Santa Clara, CA, USA) under the same HPLC conditions. Triple quadrupole tandem mass analysis was accomplished using an Agilent 6460 Triple Quadrupole LC-MS (Agilent Technologies Inc.). An electrospray ionization (ESI) interface was equipped and worked in negative ionization mode. Full scan mode was set as the mass detection mode from 100 *m*/*z* to 1000 *m*/*z*.

## 4. Conclusions

In this study, BSA bound ligands in *Radix astragali* were screened out and identified by BSA-MN coupled with HPLC-MS. The BSA-MN was prepared and characterized by TEM, XRD, FT-IR and VSM. The experiment conditions were optimized. The results revealed that there were four bound ligands from the *n*-butanol part of a *Radix astragali* extract that showed BSA affinities under the optimum conditions. In addition, the resulting reusability of BSA-MN could effectively reduce the expense of experiments. Compared with the conventional bioassay approach, this method enabled screening and identification of bound ligands from complex mixtures rapidly.

## Figures and Tables

**Table 1:** The identification, retention time, UV and MS characteristics of compounds in *Radix astragali* extract.

No.	Identification	Rt (min)	Proposed Ions (*m*/*z*)		λmax (nm)
**1**	Genistin	16.02	[M + H]^−^	433	260
			[M − glc + H]^−^	271	
**2**	Calycosin-7-*O*-β-d-glucoside	20.50	[M + H]^−^	447	260, 290
			[M − glc + H]^−^	285	
**3**	Ononin	25.01	[M + H]^−^	431	260, 310
			[M − glc + H]^−^	269	
**4**	Formononetin	33.36	[M + H]^−^	269	260, 315
			[M − CH_3_ + H]^−^	254	

## References

[1] Rodrigues T., Reker D., Schneider P., Schneider G. (2016). Counting on natural products for drug design. Nat. Chem..

[2] Lu F., Luo G., Qiao L., Jiang L., Li G., Zhang Y. (2016). Virtual Screening for Potential Allosteric Inhibitors of Cyclin-Dependent Kinase 2 from Traditional Chinese Medicine. Molecules.

[3] Xie Y., Song X., Sun X., Huang J., Zhong M., Lotze M.T., Zeh H.J., Kang R., Tang D. (2016). Identification of baicalein as a ferroptosis inhibitor by natural product library screening. Biochem. Biophys. Res. Commun..

[4] Yang Z., Wu Y., Wu S. (2016). A combination strategy for extraction and isolation of multi-component natural products by systematic two-phase solvent extraction-^13^C nuclear magnetic resonance pattern recognition and following conical counter-current chromatography separation: Podophyllotoxins and flavonoids from *Dysosma versipellis* (Hance) as examples. J. Chromatogr. A.

[5] Li S.P., Zhao J., Yang B. (2011). Strategies for quality control of Chinese medicines. J. Pharm. Biomed..

[6] Wang B.C., Deng J., Gao Y.M., Zhu L.C., He R., Xu Y.Q. (2011). The screening toolbox of bioactive substances from natural products: A review. Fitoterapia.

[7] Yang X.X., Wang Y.W., Zhang X.X., Chang R.M., Li X.N. (2011). Screening vasoconstriction inhibitors from traditional Chinese medicines using a vascular smooth muscle/cell membrane chromatography-offline-liquid chromatography-mass spectrometry. J. Sep. Sci..

[8] Zhao A., Li L., Li B., Zheng M., Tsao R. (2016). Ultrafiltration LC-ESI-MS^n^ screening of 5-lipoxygenase inhibitors from selected Chinese medicinal herbs *Saposhnikovia divaricata*, *Smilax glabra*, *Pueraria lobata* and *Carthamus tinctorius*. J. Funct. Foods.

[9] Jin Y., Cheng X., Jiang F., Guo Z., Xie J., Fu L. (2016). Application of the ultrafiltration-based LC-MS approach for screening PTP1B inhibitors from Chinese red yeast rice. Anal. Methods.

[10] Triñanes S., Casais M.C., Mejuto M.C., Cela R. (2016). Matrix solid-phase dispersion followed by liquid chromatography tandem mass spectrometry for the determination of selective ciclooxygenase-2 inhibitors in sewage sludge samples. J. Chromatogr. A.

[11] Li D.Q., Zhao J., Li S.P. (2014). High-performance liquid chromatography coupled with post-column dual-bioactivity assay for simultaneous screening of xanthine oxidase inhibitors and free radical scavengers from complex mixture. J. Chromatogr. A.

[12] Rea V., Falck D., Kool J., de Kanter F.J.J., Commandeur J.N.M., Vermeulen N.P.E., Niessen W.M.A., Honing M. (2013). Combination of biotransformation by P450 BM3 mutants with on-line post-column bioaffinity and mass spectrometric profiling as a novel strategy to diversify and characterize p38α kinase inhibitors. MedChemComm.

[13] Lu S., Luo Q., Li X., Wu J., Liu J., Xiong S., Feng Y.Q., Wang F. (2010). Inhibitor screening of protein kinases using MALDI-TOF MS combined with separation and enrichment of phosphopeptides by TiO_2_ nanoparticle deposited capillary column. Analyst.

[14] Wang E.K., Guo S.J., Li D., Zhang L.M., Li J. (2009). Monodisperse mesoporous superparamagnetic single-crystal magnetite nanoparticles for drug delivery. Biomaterials.

[15] Jiang J.S., Gan Z.F., Yang Y., Du B., Qian M., Zhang P. (2008). Immobilization of homing peptide on magnetite nanoparticles and its specificity in vitro. J. Biomed. Mater. Res. A.

[16] Ichikawa S., Kuroiwa T., Noguchi Y., Nakajima M., Sato S., Mukataka S. (2008). Production of chitosan oligosaccharides using chitosanase immobilized on amylose-coated magnetic nanoparticles. Process Biochem..

[17] Pati S.S., Singh L.H., Guimarães E.M., Mantilla J., Coaquira J.A.H., Oliveira A.C., Sharma V.K., Garg V.K. (2016). Magnetic chitosan-functionalized Fe_3_O_4_@Au nanoparticles: Synthesis and characterization. J. Alloys Compd..

[18] Atacan K., Çakıroğlu B., Özacar M. (2016). Improvement of the stability and activity of immobilized trypsin on modified Fe_3_O_4_ magnetic nanoparticles for hydrolysis of bovine serum albumin and its application in the bovine milk. Food Chem..

[19] Kim S., Lee J., Jang S., Lee H., Sung D., Chang J.H. (2016). High efficient chromogenic catalysis of tetramethylbenzidine with horseradish peroxidase immobilized magnetic nanoparticles. Biochem. Eng. J..

[20] Casado-Carmona F.A., del Alcudia-León M.C., Lucena R., Cárdenas S., Valcárcel M. (2016). Magnetic nanoparticles coated with ionic liquid for the extraction of endocrine disrupting compounds from waters. Microchem. J..

[21] Cao Y., Wen L., Svec F., Tan T., Lv Y. (2016). Magnetic AuNP@Fe_3_O_4_ nanoparticles as reusable carriers for reversible enzyme immobilization. Chem. Eng. J..

[22] Siurdyban E., Brotin T., Talaga D., Heuzé K., Vellutini L., Buffeteau T. (2016). Immobilization of Cryptophane Derivatives onto γ-Fe_2_O_3_ Core–Shell Magnetic Nanoparticles. J. Phys. Chem. C.

[23] Larsen G.K., Farr W., Hunyadi Murph S.E. (2016). Multifunctional Fe_2_O_3_–Au Nanoparticles with Different Shapes: Enhanced Catalysis, Photothermal Effects, and Magnetic Recyclability. J. Phys. Chem. C.

[24] Nazari Serenjeh F., Hashemi P., Naeimi H., Zakerzadeh E., Ghiasvand A.R. (2016). Spherical agarose-coated magnetic nanoparticles functionalized with a new salen for magnetic solid-phase extraction of uranyl ion. Microchim. Acta.

[25] Tao Y., Chen Z., Zhang Y., Wang Y., Cheng Y. (2013). Immobilized magnetic beads based multi-target affinity selection coupled with high performance liquid chromatography–mass spectrometry for screening anti-diabetic compounds from a Chinese medicine “Tang-Zhi-Qing”. J. Pharm. Biomed..

[26] Tao Y., Zhang Y., Cheng Y., Wang Y. (2013). Rapid screening and identification of α-glucosidase inhibitors from mulberry leaves using enzyme-immobilized magnetic beads coupled with HPLC/MS and NMR. Biomed. Chromatogr..

[27] Chu C., Qi L.W., Liu E.H., Li B., Gao W., Li P. (2010). *Radix astragali* (Astragalus): Latest Advancements and Trends in Chemistry, Analysis, Pharmacology and Pharmacokinetics. Curr. Org. Chem..

[28] Luo Y., Qin Z., Hong Z., Zhang X., Ding D., Fu J.H., Zhang W.D., Chen J. (2004). Astragaloside IV protects against ischemic brain injury in a murine model of transient focal ischemia. Neurosci. Lett..

[29] Gui S.Y., Wei W., Wang H., Wu L., Sun W.Y., Chen W.B., Wu C.Y. (2006). Effects and mechanisms of crude astragalosides fraction on liver fibrosis in rats. J. Ethnopharmacol..

[30] Wang H., Li J., Yu L., Zhao Y., Ding W. (2004). Antifibrotic effect of the Chinese herbs, Astragalus mongholicus and Angelica sinensis, in a rat model of chronic puromycin aminonucleoside nephrosis. Life Sci..

[31] Qi L.W., Cao J., Li P., Yu Q.T., Wen X.D., Wang Y.X., Li C.Y., Bao K.D., Ge X.X., Cheng X.L. (2008). Qualitative and quantitative analysis of *Radix astragali* products by fast high-performance liquid chromatography-diode array detection coupled with time-of-flight mass spectrometry through dynamic adjustment of fragmentor voltage. J. Chromatogr. A.

[32] Soares S., Mateus N., de Freitas V. (2007). Interaction of Different Polyphenols with Bovine Serum Albumin (BSA) and Human Salivary α-Amylase (HSA) by Fluorescence Quenching. J. Agric. Food Chem..

[33] Arroyo-Maya I.J., Campos-Terán J., Hernández-Arana A., McClements D.J. (2016). Characterization of flavonoid-protein interactions using fluorescence spectroscopy: Binding of pelargonidin to dairy proteins. Food Chem..

[34] Tian Z.Y., Song L.N., Zhao Y., Zang F.L., Zhao Z.H., Chen N.H., Xu X.J., Wang C.J. (2015). Spectroscopic Study on the Interaction between Naphthalimide-Polyamine Conjugates and Bovine Serum Albumin (BSA). Molecules.

[35] Li Y., Xu X., Deng C., Yang P., Zhang X. (2007). Immobilization of Trypsin on Superparamagnetic Nanoparticles for Rapid and Effective Proteolysis. J. Proteome Res..

[36] Liu L., Ma Y., Chen X., Xiong X., Shi S. (2012). Screening and identification of BSA bound ligands from Puerariae lobata flower by BSA functionalized Fe_3_O_4_ magnetic nanoparticles coupled with HPLC-MS/MS. J. Chromatogr. B.

[37] Mahesh M., Arivizhivendhan K.V., Maharaja P., Boopathy R., Hamsavathani V., Sekaran G. (2016). Production, purification and immobilization of pectinase from *Aspergillus ibericus* onto functionalized nanoporous activated carbon (FNAC) and its application on treatment of pectin containing wastewater. J. Mol. Catal. B Enzym..

[38] Ozseker E.E., Akkaya A. (2016). Development of a new antibacterial biomaterial by tetracycline immobilization on calcium-alginate beads. Carbohydr. Polym..

[39] Waifalkar P.P., Parit S.B., Chougale A.D., Sahoo S.C., Patil P.S., Patil P.B. (2016). Immobilization of invertase on chitosan coated γ-Fe_2_O_3_ magnetic nanoparticles to facilitate magnetic separation. J. Colloid Interface Sci..

[40] Golfam G., Karen C.W. (2016). Capillary Electrophoretic Peptide Mapping to Probe the Immobilization/Digestion Conditions of Glutaraldehyde-crosslinked Chymotrypsin. Curr. Anal. Chem..

[41] Rehman S., Bhatti H.N., Bilal M., Asgher M. (2016). Cross-linked enzyme aggregates (CLEAs) of *Pencilluim notatum* lipase enzyme with improved activity, stability and reusability characteristics. Int. J. Biol. Macromol..

[42] Aybaster O., Sahin S., Isk E., Demir C. (2011). Determination of total phenolic content in *Prunella* L. by horseradish peroxidase immobilized onto chitosan beads. Anal. Methods.

[43] Kauldhar B.S., Dhau J.S., Sooch B.S. (2016). Covalent linkage of alkalothermophilic catalase onto functionalized cellulose. RSC Adv..

[44] Jian H., Wang Y., Bai Y., Li R., Gao R. (2016). Site-Specific, Covalent Immobilization of Dehalogenase ST2570 Catalyzed by Formylglycine-Generating Enzymes and Its Application in Batch and Semi-Continuous Flow Reactors. Molecules.

[45] Shi Q., Chen J., Wang Y., Li Z., Li X., Sun C., Zheng L. (2015). Immobilization of Cyclooxygenase-2 on Silica Gel Microspheres: Optimization and Characterization. Molecules.

[46] Tang Y., Li S., Li S., Yang X., Qin Y., Zhang Y., Liu C. (2016). Screening and isolation of potential lactate dehydrogenase inhibitors from five Chinese medicinal herbs: Soybean, *Radix pueraria*, *Flos pueraria*, *Rhizoma belamcandae*, and *Radix astragali*. J. Sep. Sci..

[47] Shi J., Zheng L., Lin Z., Hou C., Liu W., Yan T., Zhu L., Wang Y., Lu L., Liu Z. (2015). Study of pharmacokinetic profiles and characteristics of active components and their metabolites in rat plasma following oral administration of the water extract of *Astragali radix* using UPLC-MS/MS. J. Ethnopharmacol..

[48] Pan H., Li X., Cheng X., Wang X., Fang C., Zhou T., Chen J. (2015). Evidence of calycosin-7-*O*-β-d-glucoside’ role as a major antioxidant molecule of *Astragalus membranaceus* Bge. var. *mongholicus* (Bge.) Hsiao plants under freezing stress. Environ. Exp. Bot..

[49] Liu X.H., Zhao J.B., Guo L., Yang Y.L., Hu F., Zhu R.J., Feng S.L. (2014). Simultaneous Determination of Calycosin-7-*O*-β-d-Glucoside, Ononin, Calycosin, Formononetin, Astragaloside IV, and Astragaloside II in Rat Plasma After Oral Administration of *Radix astragali* Extraction for Their Pharmacokinetic Studies by Ultra-Pressure Liquid Chromatography with Tandem Mass Spectrometry. Cell Biochem. Biophys..

[50] Fu S., Gu Y., Jiang J.Q., Chen X., Xu M., Chen X., Shen J. (2014). Calycosin-7-*O*-β-d-glucoside regulates nitric oxide /caveolin-1/matrix metalloproteinases pathway and protects blood–brain barrier integrity in experimental cerebral ischemia–reperfusion injury. J. Ethnopharmacol..

[51] Xiao J.B., Wu M., Kai G., Wang F., Cao H., Yu X. (2011). ZnO-ZnS QDs interfacial heterostructure for drug and food delivery application: enhancement of the binding affinities of flavonoid aglycones to bovine serum albumin. Nanomed. Nanotechnol..

[52] Liu E.H., Qi L.W., Li P. (2010). Structural Relationship and Binding Mechanisms of Five Flavonoids with Bovine Serum Albumin. Molecules.

[53] Wen X.D., Qi L.W., Chen J., Song Y., Yi L., Yang X.W., Li P. (2007). Analysis of interaction property of bioactive components in Danggui Buxue Decoction with protein by microdialysis coupled with HPLC–DAD–MS. J. Chromatogr. B.

